# Terpenes from *Cecropia* Species and Their Pharmacological Potential

**DOI:** 10.3390/ph17030399

**Published:** 2024-03-20

**Authors:** Preslav Enchev, Yancho Zarev, Anzhelica Dakovska, Andrés Rivera-Mondragón, Ekaterina Kozuharova, Iliana Ionkova

**Affiliations:** 1 Department of Pharmacognosy, Faculty of Pharmacy, Medical University of Sofia, 2 Dunav Str., 1000 Sofia, Bulgaria; p.enchev@pharmfac.mu-sofia.bg (P.E.); angelicadakovska98@gmail.com (A.D.); ionkova@pharmfac.nat.bg (I.I.); 2 Instituto Especializado de Análisis (IEA), Universidad de Panamá, Ciudad de Panama P.O. Box 3366, Panama; andres.riveraa@up.ac.pa; 3 Centro de Investigaciones Farmacognósticas de la Flora Panameña (CIFLORPAN), Departamento de Química Medicinal y Farmacognosia, Facultad de Farmacia, Universidad de Panamá, Ciudad de Panama P.O. Box 3366, Panama

**Keywords:** *Cecropia* genus, terpene source, pharmacology, medicinal use

## Abstract

*Cecropia* is a genus of neotropical trees mainly distributed in Mexico and Central and South America. Currently, 63 species have been described, some of which have been used for centuries in traditional medicine to treat conditions such as diabetes, high blood pressure, and wound healing, among others. In recent times, modern phytochemical studies have succeeded in isolating individual compounds with potential specific medicinal applications. This review aims to examine the literature data regarding isolated terpenes and their correlation with pharmacological activities, with the goal of unveiling the future potential of the genus.

## 1. Introduction

The genus *Cecropia* Loefl. (Urticaceae) represents the largest genus of the family *Cecropiaceae*, with 61 described species [1]. By 2023, the number of identified species has increased to 63 [2]. *Cecropia*’s natural habitat encompasses tropical and subtropical rainforests, up to altitudes of 2600 m, across Mexico to South America [2]. Typically, *Cecropia* species are trees of secondary vegetation, ranging from 5 to 25 m tall, with a sectioned stem, upright, hollow, and broad leaves with unique hues and textures [1]. The trees are fast-growing and abundantly found in their native habitat [2]. They are primary colonizers of deforested areas in the tropics [3], and *C. peltata* is considered as one of the “100 of the World’s Worst Invasive Alien Species” by the Global Invasive Species Database (2005) and has a “high-risk” status and a score of 9 by the Pacific Island Ecosystems at Risk [4]. *Cecropia* trees are known vernacularly as “guarumo”, “guarumbo”, “yarumo”, “embaúba”, “ambay”, “trum-pet tree”, and “torém” [5,6,7,8]. These popular names can change depending on the color of the flowers, for example, embaúba vermelha (red embaúba, *C. glaziovii*), embaúba-branca (white-embaúba, *C. palmata*), and embaúba-prateada (gray embaúba, *C. pachystachya*) [9]. Moreover, a majority of the species within the genus *Cecropia* are recognized as myrmecophytes or ant-plants [10]. They often engage in mutualistic interactions with specific ant colonies, predominantly with those from the *Azteca* genus [11], which is beneficial for both organisms [12,13,14]. The *Cecropia* genus is recognized in ethnopharmacology, though many of its constituents remain undescribed. Our goal is to provide insights into our current knowledge to explore future opportunities for modern research.

## 2. Ethnopharmacological Aspects of the Genus *Cecropia*

Ethnopharmacology bridges the gap between traditional medicinal plants’ uses and modern drug discovery. So, it plays important roles in drug discovery, in the understanding of phytochemistry and the significance of traditional knowledge, and in the industry’s shift towards natural product research [15]. In this context, *C. hololeuca* Miquel. (commonly known as ‘imbaúba’) is highlighted, as it is included in the First Edition of the Brazilian Pharmacopeia [16]. Similarly, *C. adenopus* Mart. (‘ambay’) is listed in the Argentinian Pharmacopeia (6th edition) [17]. In addition to this, these species have a wide range of traditional uses [18]. In El Salvador, the leaves of *C. obtusifolia* are employed to make a sedative tea, and are used in treating conditions such as arthritis, rheumatism, and inflammations [19]. This same species is utilized to treat cardiovascular diseases in Panama [20] and in Mexico for conditions like cough, asthma, bronchitis, fever, hepatic and kidney diseases, rheumatism, and inflammation, among others, as well as for its diuretic effect [21]. Another frequently utilized species in traditional medicine is *C. peltata*, whose leaves are primarily used to treat cough, respiratory problems, blood and circulatory system disorders, hypertension, diabetes, and kidney infections and to promote kidney functions [18]. One recurring use of the *Cecropia* genus across different countries in South America is its application in lowering blood sugar levels and preventing type 2 diabetes. Local communities use the leaves and bark of *C. hololeuca*, *C. peltata*, *C. palmata*, and *C. obtusa* through oral administration in Brazil [22]. Similarly, *C. glaziovii* leaves are used in Colombia [23], as are the leaves, bark, and roots of the endemic species *C. mutisiana* [24]. The widespread use of *C. obtusifolia* in Mexico has sparked an interest among researchers to further investigate its anti-diabetic activity [21,25,26,27]. Another species with traditional use for diabetes in folk medicine is *C. pachystachya* [28]. The leaves of this plant are boiled in water and the infusion is drunk throughout the day [29]. Other common ethnopharmacological uses for the genus include treatments for inflammation, respiratory conditions like asthma and cough, heart diseases, and high blood pressure [18]. While the genus has numerous ethnopharmacological applications, 14 species are specifically noted for their significant use [18].

The objective of this review is to provide a detailed analysis of the genus *Cecropia*, with a particular focus on its terpenes and their associated pharmacological activities. Terpenes, known for their diverse biological activities [30,31], are integral to our understanding of the therapeutic potential of this genus. By delving into the chemical constituents, especially terpenes, we aim to highlight possibilities for establishing and conducting targeted phytochemical assays on *Cecropia* species that remain underexplored. This proactive approach is imperative, given the burgeoning interest in natural products among researchers [15]. Thus, we hope to ensure that the therapeutic potential of these species is not overlooked and that its full pharmacological potential is realized. The literature data used in this review are primarily from the last 15 years, although information from the last 40 years is also included.

## 3. Terpenes Identified from the Genus *Cecropia*

### 3.1. Importance of Terpenes

**Figure 1:**
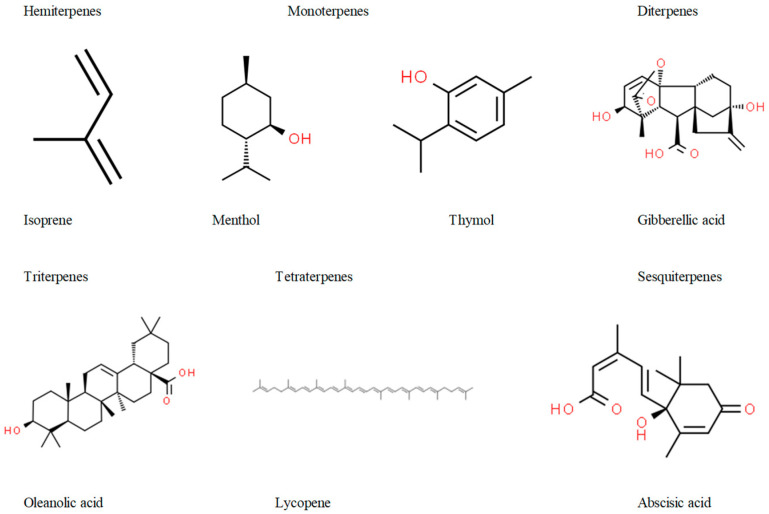
General classification of terpenes.

Terpenes, also known as isoprenoids, are the most expansive and diverse class of natural compounds predominantly found in plants but also certain animals [32]. Essential to the evolution and survival of plants, they play critical roles in plant defense, signaling, climatic acclimation, and more, while also being responsible for the unique fragrances, flavors, and pigments in flora [32,33]. Beyond their ecological functions, terpenes have found myriad applications as pharmaceuticals, as well as in other industrial applications, such as in the biofuel sector [33], reflecting their vast chemical diversity and status as high-value bioproducts. Rooted in both the primary and secondary metabolism, these compounds, constructed from repetitive five-carbon units of isopentenyl diphosphate (IPP), remain substantially unexplored, despite comprising approximately 40,000 known structures [30,32,34]. Depending on the number of isoprene units, terpenes can be classified into mono-, di-, tri-, tetra-, and sesquiterpenes (Figure 1).

Triterpenes are a broad and varied group of terpenes that originate from two C15 units, forming 30-carbon precursors like squalene. Their cyclization and oxidation create diverse structures. These transformations lead to tetra- and pentacyclic triterpenes or, through cycloartenole, to products like cucurbitacines, cholesterol, phytosterols, and steroid saponins. Typical triterpene structures encompass pentacyclic forms like oleanane and lupine and tetracyclic types like dammarane [35,36].

From a pharmacological perspective, terpenes are well known for their anti-plasmodial, anti-viral, anti-depressant, anti-diabetic, and anti-cancer activities [30]. Moreover, recent studies have underscored their emerging potential in the field of inflammation, addressing challenges faced by current inflammatory treatments [37,38,39]. Terpenes are widely acclaimed for their anti-cancer activity. For instance, an early 1997 study concluded that a combination of monoterpenes, diterpenes, and sesquiterpenes can be effectively used to treat cancers that occur in the colon, brain, prostate gland, and bones [40]. It also claimed that the administration of terpenes in humans inhibited the growth of prostate cancer cells and sensitized the tumor in such a way that it became susceptible to radiotherapy [30]. The major advantage of this treatment was that the drug can be administered through several routes, among which oral and topical were most preferred [30]. Terpenes exhibit potential anti-cancer activity through various mechanisms such as stalling cancer cell proliferation, inducing apoptosis, and inhibiting angiogenesis. One notable example is limonene, which recent studies have identified as a potential chemopreventive and chemotherapeutic agent against various cancers, including those of the colon, brain, and prostate gland [30]. Additionally, terpenes like taxol and phorbol have already made their mark in the therapeutic domain, being integral components of certain anti-cancer drugs, underscoring the importance of this class of organic compounds in medicinal chemistry [41]. Tanshinones, derived from the traditional Chinese herb *Salvia miltiorrhiza* (often referred to as Danshen or Tanshen), have also been spotlighted for their potent anti-cancer properties, both in vitro and in vivo [42]. These compounds have a broad range of anti-cancer activities, from anti-proliferation to inhibiting adhesion, migration, and invasion. Owing to these properties, the synthesis of tanshinone analogues has been the subject of many clinical investigations [43,44]. Beyond their anti-cancer attributes, tanshinones are believed to reduce inflammation and amplify immune responses, and they have historically found use in many Asian countries as remedies for ailments ranging from heart diseases to arthritis [42]. On a different note, the diterpene alcohols cafestol and kahweol, predominant in coffee beans, are associated with several health benefits [30]. Coffee consumption has been linked to reduced risks of conditions such as depression, prostate cancer, stroke, diabetes, and some cancers, with the anti-inflammatory and antioxidant properties of these diterpenes thought to play a crucial role [30]. In essence, the terpenes abundant in various plants and dietary sources present promising avenues for research and potential inclusion in anti-cancer therapeutic regimens [30]. Pentacyclic triterpenes, specifically maslinic and oleanolic acid, possess antioxidant properties, modulate the immune system, and offer protection against liver disease, peptic ulcer disease, HIV, cancer, diabetes, hyperlipidemia, and atherosclerosis [45]. Specifically, these naturally occurring and virtually non-toxic compounds can reduce serum glucose and lipids, potentially serving as novel treatments for type 2 diabetes mellitus, hyperlipidemia, and atherosclerosis [45]. Ursolic acid (UA) and its derivatives have garnered attention due to their promising pharmacological activities against non-communicable diseases (NCDs) like cancer and diabetes. With its origins traced to medicinal plants, UA boasts a spectrum of biological effects, including anti-inflammatory, anti-cancer, anti-diabetic, and antioxidant properties, but challenges in terms of its bioavailability and solubility have posed limitations to its clinical applications [46]. Recent advances have identified strategies to modify UA’s molecular structure, focusing on its main active sites, to enhance its anti-cancer capabilities [47]. A notable derivative, US597, demonstrated potential in suppressing cancer cells adhesion, migration, and invasion, showing encouraging outcomes in melanoma lung metastasis studies [48]. Moreover, UA’s broad range of biological activities encompasses potential health benefits in inflammation, metabolic diseases, cardiovascular diseases, and neurological disorders, with the discovery of intriguing analogs like 23-hydroxy ursolic acid [49]. Furthermore, UA’s derivatives exhibit significant improvement in anti-proliferative activity against various cancer cell lines, underscoring the promise of structural modification to elevate their efficacy [47]. Finally, UA and its analogs stand out as potent therapeutic agents for cancer, diabetic neuropathy, and inflammatory diseases, offering a multi-target approach to tackling these ailments [50]. The terpenes found in *Cecropia* species are tricyclic and pentacyclic triterpenes of the oleanane and ursane types (Table 1).

### 3.2. Methods for Terpenes Isolation from Cecropia Species

Roots of *C. lyratiloba* weighing 1.30 kg were processed and extracted with methanol. This methanolic extract was further partitioned through a series of solvents: hexane, dichloromethane, ethyl acetate, and butanol. From these, the dichloromethane fraction (8.18 g) was specially treated with silica gel column chromatography, adopting chloroform and ethyl acetate as the solvents of choice in a polarity gradient. This process pinpointed a fraction rich in triterpenes, recognized via its TLC profile. The solvent system’s empirical screening was conducted using the shake flask method, where the triterpene mixture’s distribution in a hexane–ethyl acetate–methanol–water solvent system was examined. Based on the results, five different compositions of this solvent system were prepared and used to treat the triterpene mixture. The mixture’s solute partitioning was then visualized via TLC plates. To separate the triterpenes, a two-step chromatographic process was adopted. In this initial separation, a certain procedure was followed, including specific rpm, flow rates, and solvent systems. Following this, one of the fractions with a weight of 253 mg was identified to contain a triterpene mixture. This fraction was further subjected to another round of chromatography with an optimized solvent gradient, leading to the isolation of specific compounds. This refined approach, utilizing high-speed countercurrent chromatography, was not effective at separating the triterpenes using an isocratic system. As a solution, a gradient system was designed by adjusting the methanol content in the solvent mixture. The methanol gradient in the solvent system played a pivotal role in separating the triterpenes based on their subtle differences in partition coefficients. The HSCCC experiment used sequential solvent systems, and the results led to the isolation of tormentic acid and a mixture of tormentic acid and euscaphic acids. These compounds were identified using ^13^C-NMR data and literature comparisons. For further refinement and separation of the triterpenes present in a specific fraction, a second HSCCC run was executed. By optimizing the methanol ratio in the solvent system, several compounds were isolated and identified through ^13^C-NMR data. Among these were mixtures of tormentic and isoarjunolic acids, euscaphic acid, and 3-acetyl-tormentic acid [54].

**Figure 2:**
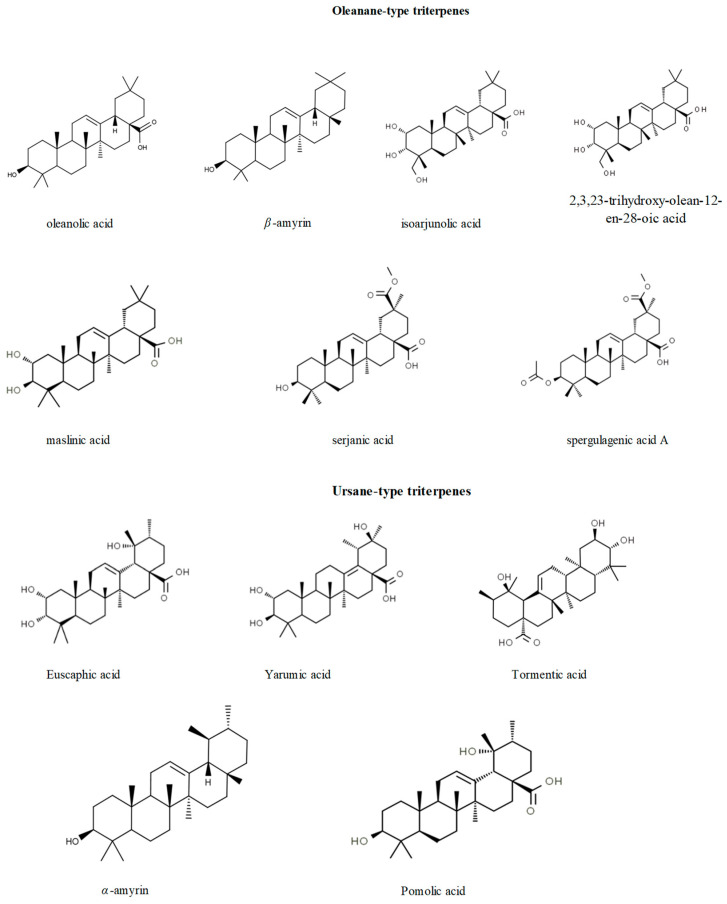
The chemical structures of the main isolated terpenes from *Cecropia* species.

Barks sourced from *C. lyratiloba* underwent a thorough drying and crushing process before extraction with methanol. To further isolate specific compounds, the resulting methanol extract was first dissolved in a 50%methanol/water mixture. This mixture then underwent successive partitioning using solvents of varied polarities: hexane, dichloromethane, ethyl acetate, and butanol. The dichloromethane fraction was subjected to countercurrent chromatography. For the separation, a gradient of solvent systems was employed, starting from a less polar solvent system and transitioning to more polar ones. This encompassed a mix of hexane, ethyl acetate, methanol, and water in varying ratios. The method involved a rotation technique, the injection of the dichloromethane fraction, and the subsequent collection of eluted fractions. With the completion of one solvent system, the mobile phase was promptly transitioned to the next, more polar system. As a result of the chromatographic separation and subsequent spectroscopic analysis, the identified compounds from the *C. lyratiloba* bark extract were euscaphic acid, tormentic acid, and 2*α*- and 3*β*-acetyl tormentic acids [53]. The structures of the compounds are shown in Figure 2.

*C. catharinensis* (600 g) of air-dried powdered roots underwent a successive extraction using hexane, CH_2_Cl_2_, and ethyl acetate. The extraction process included purification using column chromatography on silica gel and various gradient methods, with preparative TLC (hexane–ethyl acetate 7:3), further refining the hexane extract. From these procedures, the following compounds were isolated: *β*-sitosterol (320 mg), 2-*O*-acetyl-tormentic acid (48 mg), 2-*O*-acetyl euscaphic acid (30 mg), tormentic acid (82 mg), and mixtures like *β*-sitosterol-60-n-acyl)-glycosides (120 mg). The extended purification of various extracts also yielded other compounds such as euscaphic, pomolic, and 2,3,23,-trihydroxy-olean-12-en-28-oic acids [58]. Pomolic acid was derived from the dry and powdered aerial parts of *C. pachystachya*, extracted using dichloromethane in a Soxhlet apparatus and subsequently isolated through a series of chromatographic processes, as confirmed by ^1^H-NMR [57].

The roots of *C. telenitida* were sourced from la Ceja, Antioquia, and authenticated using a deposited specimen at the Universidad de Antioquia herbarium. After air-drying, their roots (2 kg) underwent a cleaning process using *n*-hexane and ethanol. Subsequent extraction with ethyl acetate produced an extract that was concentrated to yield 112 g of material. This extract was diluted in methanol and subjected to purification by Sephadex LH-20 using medium-pressure liquid chromatography. This step aimed to remove high-molecular-weight molecules such as polymers. The resulting fractions, identified to contain triterpenes through the sulfuric acid/anisaldehyde reagent, yielded about 1% and were combined to produce 20 g of material, which was then further analyzed and used for various assays. Conventional preparative HPLC techniques were used to isolate and purify the triterpene molecules. The primary triterpenes identified in significant amounts from *C. telenitida* roots were serjanic acid and spergulagenic acid A, while yarumic acid was found in lesser amounts. Other triterpenes, namely 20-hydroxy-ursolic acid and goreishic acid I, were detected in trace amounts of less than 5 mg. All these compounds were confirmed and their structures characterized through various spectral analyses [8].

Solvents of varying polarity, including n-hexane and ethyl acetate/methanol, were utilized for extraction using a solid–liquid semi-pilot extraction device with controlled stirring rate and extraction time. After the extraction, the samples underwent fractionation using an Isolera flash purification system, with each extract divided into 20–25 fractions, dried under high vacuum, and chemically characterized using thin-layer chromatography with a standard protocol to the isolation of isoyarumic acid [55].

Fresh plant specimens of *C. obtusa* and *C. palmata* were dried, ground into a fine powder, and subjected to ultrasound-assisted extraction using ethyl acetate or chloroform for 30 min at 37C. Among the triterpenes separated were *α*-amirin, *β*-amirin, lupeol, maslinic, and oleanolic acids for both species, as well as erythrodiol in *C. palmata* [51]. Notably, ethyl acetate was found to be a more efficient extraction solvent for certain compounds than chloroform. Post-extraction, HPLC analysis revealed that the best mobile phase was ACN/THF (90:10; *v*/*v*) with an optimal detection wavelength at 210 nm. The triterpenes were detected as major compounds in the extracts, reaffirming the pharmacological significance of such plants in folk medicine. The developed HPLC method was effective at separating and quantifying these compounds, showing its applicability in medicinal plant research and in the quality control of herbal medicines [51].

Four *Cecropia* species—*C. peltata*, *C. obtusa*, *C. hispidissima*, *C. insignis*—underwent phytochemical analysis, where the leaves were dried, ground, cleaned with n-hexane, macerated in 70% ethanol, and filtered. In the course of a more detailed phytochemical investigation, the dried ethanol extract was reconstituted in water and subjected to MCI gel chromatography. Selected fractions, characterized by analogous TLC patterns, were pooled and then passed through a silica gel column. Subsequent separation was carried out using Sephadex LH-20 and eluted with methanol. The final isolation of the pentacyclic triterpenes euscaphic acid 28-*O*-glucoside (Kaji-ichigoside F1), triterpenoid saponin-*O*-hexoside, and tormentic acid 28-*O*-glucoside (tormentoside) and the inseparable mixture of niga-ichigoside F2 and buergericic acid 28-*O*-glucoside was achieved through semi-preparative HPLC (C_18_) employing a solvent system of water + FA 0.1% and acetonitrile + FA 0.1% [52].

Roots from *C. telenitida* were collected from Antioquia in Colombia and dried and ground into a fine powder. Then, the material was subjected to an extraction process using a solvent system of dichloromethane and ethyl acetate. This extraction was conducted at room temperature and under constant stirring for 14 days in a specially designed semi-pilot extraction system. Following the extraction, the fractions obtained underwent a further purification process using a preparative HPLC. This stage of separation was monitored at a wavelength of 210 nm and utilized a gradient elution method. The elution involved a transition from a 40% acetonitrile solution, gradually increasing to 95%. This process led to the successful isolation and identification of several pentacyclic triterpenes from the *C. telenitida* roots. Among the compounds isolated were isoyarumic acid, tormentic acid, arjunolic acid, and hederagenic acid [56].

Even those structures previously confirmed by 1D NMR experiments (^1^H and ^13^C) for complete structure elucidation are nowadays strictly required to undergo 2D NMR (COSY, HSQC and HMBC) experiments.

### 3.3. Cecropia Terpenes’ Pharmacology

The roots of *C. lyratiloba* were found to be natural sources of four distinct triterpenoids, including euscaphic acid and tormentic acid, along with their acetylated derivatives. Notably, these compounds have exhibited in vitro cytotoxicity against sensitive leukemia cell lines (K562), even those that are multidrug-resistant (Lucena 1). Furthermore, euscaphic acid has been observed to induce caspase-dependent apoptosis, emphasizing its potential utility in treating various neoplasias, particularly those with the MDR phenotype (52). *C. telenitida* is traditionally used in Latin America as a remedy for metabolic disorders and diabetes. The roots of this species contain pentacyclic triterpenes that display a hypoglycemic effect. A specific fraction from a chemical library derived from these roots was able to inhibit the 11*β*-hydroxysteroid dehydrogenase type 1 enzyme by 82%. This enzyme is responsible for converting cortisone to cortisol. Within this fraction, a molecule with an IC_50_ of 0.95 ± 0.009 µM, isolated and identified as isoyarumic acid, confirmed the inhibitory role of the pentacyclic triterpene, bolstering its potential in combatting metabolic disorders [55]. In addition, *C. telenitida*’s traditional medicinal use is supported by the presence of pentacyclic triterpenes with oleanane and ursane scaffolds that are implicated in preventing type 2 diabetes. Furthermore, specific triterpenoids, including lupeol, oleanolic acid, and ursolic acid, are products of this plant’s unique biosynthethic pathway [56]. Another study examined the triterpene-enriched fraction (TEF) from the leaves of *C. pachystachya* for their cytotoxic effects on cancer cells. The results revealed TEF did influence prostate PC3 cancer cells, inducing senescence without triggering apoptosis. Interestingly, these effects were seen in the cancer cells but not in the non-tumor counterparts. TEF treatment also curtailed the duplication capacity of these cells. This investigation underscores the potential of terpenes, against a spectrum of cancers, including prostate cancer. Some triterpenoids, including ursolic acid, have advanced to clinical trials due to their role in treatment resistance during cancer recurrence are areas needing further exploration [59].

Specifically, a pentacyclic triterpene fraction from *C. telenitida* Cuatrec., Urticaceae roots was analyzed. The study identified a novel compound termed yarumic acid, along with four known molecules (serjanic acid, spergulagenic acid A, 20-hydroxy-ursolic acid, and goreishic acid I). These compounds were found to inhibit the secretion of proinflammatory cytokines (IL-1E, IL-12p40, IL-12p70, TNF-D) in dendritic cell (DC)-based assays. Notably, spergulagenic acid A also inhibited nitric oxide production in lipopolysaccharide-stimulated dendritic cells. The most abundant compounds, serjanic acid and spergulagenic acid A, showed the highest activity, suggesting their crucial role in the anti-inflammatory effects observed. Further research is warranted to elucidate the molecular mechanisms underlying these effects, potentially involving pathways such as NF-κB signaling triggered by Toll-like receptor 4 (TLR4) activation by lipopolysaccharide in DCs. The study suggests that these pentacyclic triterpenes, especially serjanic acid and spergulagenic acid A, contribute significantly to the anti-inflammatory properties of *C. telenitida*. Furthermore, yarumic acid and the identified pentacyclic triterpenes show promise as potential immunomodulatory and anti-inflammatory agents [8]

## 4. Discussion

The genus *Cecropia*, with its 63 described species, represents a large field of unexplored phytochemical and pharmacological potential. While the current research has primarily focused on a dozen species, most *Cecropia* species remain uninvestigated. Given the pharmacological activities of the terpenes identified so far, ranging from anti-diabetic to anti-cancer properties, it is reasonable to assume that other species within this genus may contain compounds with similar structures and pharmacological activities, which can complete our understanding of terpenes’ structures and related pharmacological actions.

Valuable ethnopharmacological data have been documented, particularly the traditional uses of *Cecropia* in treating conditions like diabetes, hypertension, and various inflammatory disorders. This traditional knowledge guides modern scientific research, suggesting specific pathways and therapeutic targets for these compounds. The discovery of terpenes, such as euscaphic acid, tormentic acid, and their derivatives in *Cecropia* species, with potent bioactivities including cytotoxic effects against cancer cells and hypoglycemic effects, prove the relevance of these species in drug development. Moreover, the potential applications of *Cecropia*’s terpenes in treating as well as in preventing metabolic disorders are especially promising. The inhibitory effects of these compounds on enzymes like the 11*β*-hydroxysteroid dehydrogenase type 1, crucial in metabolic regulation, highlight their possible role in managing conditions like type 2 diabetes. While recent studies validate certain ethnopharmacological effects of the plant by confirming the pharmacological actions of its isolated molecules, some effects still remain unverified due to limited contemporary research.

## 5. Conclusions

In conclusion, this review on the genus *Cecropia* shows that most of the phytochemical and pharmacological potential of this diverse group of neotropical trees is still not fully revealed. Therefore, future research could focus on a more comprehensive exploration of the *Cecropia* genus. This includes targeted phytochemical studies on the uninvestigated species, pharmacological testing of isolated compounds, and the development of new therapeutic agents based on these natural compounds. Additionally, *C. peltata* is a prospective source of valuable terpenes, and their use for medicinal purposes can be a factor for the control of this species, which recently has turned out to be alien invasive plant in several tropical regions. Based on these insights, there is a clear need for more thorough research on the lesser-known *Cecropia* species. The goal is to fully understand and utilize the range of pharmacological effects offered by *Cecropia*’s terpenes and to consider how they can be incorporated into new treatments for various diseases. Furthermore, while this review focuses specifically on terpenes, the *Cecropia* genus could also provide beneficial molecules from different classes of compounds.

## Figures and Tables

**Table 1:** Tentatively identified and isolated terpenes in *Cecropia* species.

Compounds	*Cecropia* Species	Ref.
Tentatively identified terpenes		
serjanic acid, spergulagenic acid A, goreishic acid I, 20-hydroxy-ursolic acid, yarumic acid	*C. telenitida*	[8]
oleanolic acid, maslinic acid, *α*-amirin, *β*-amirin, lupeol	*C. obtusa, C.palmata*	[51]
erythrodiol	*C. palmata*	[51]
euscaphic acid 28-*O*-glucoside (kaji-ichigoside F1), triterpenoid saponin-*O*-hexoside niga-ichigoside F2 and buergericic acid, 28-*O*-glucoside inseparable mixture. tormentic acid 28-*O*-glucoside (tormentoside)	*C. peltata, C. insignis, C. hispidissima, C. obtusifolia*	[52]
Isolated terpenes		
euscaphic acid, tormentic acid, 2*α*-acetyl tormentic acid, 3*β*-acetyl tormentic acid	*C. lyratiloba*	[53]
tormentic acid, euscaphic acid, isoarjunolic acid, 3-acetyl tormentic acid	*C. lyratiloba*	[54]
isoyarumic acid	*C. telenitida*	[55]
arjunolic acid, isoyarumic acid, tormentic acid, hederagenic acid	*C. telenitida*	[56]
pomolic acid	*C. pachystachia*	[57]
maslinic acid, oleanolic acid, ursolic acid, pomolic acid, tormentic acid, 2-*O*-acetyl-tormentic acid, 2*α*,3*β*,19*α*,-trihidroxy-11*α*,12α,-epoxy-ursane-28,13*β*-olide, euscaphic acid, 2-*O*-acetyl-euscaphic acid	*C. catharinensis*	[58]

## Data Availability

Data are available by request from the corresponding author ina_kozuharova@yahoo.co.uk.
